# Unilateral ovarian and fallopian tube agenesis in an infertile patient with a normal uterus

**DOI:** 10.3892/etm.2014.1825

**Published:** 2014-07-04

**Authors:** BINGYA CHEN, CHUNBO YANG, ZAYD SAHEBALLY, HANGMEI JIN

**Affiliations:** Department of Gynecology, Women’s Hospital, School of Medicine, Zhejiang University, Hangzhou, Zhejiang 310006, P.R. China

**Keywords:** unilateral adnexal agenesis, fallopian tube, ovary, congenital anomaly, primary infertility

## Abstract

Congenital agenesis of the unilateral adnexa is a condition that has rarely been described in the literature. The current study presents the case of a 26-year-old female who was admitted to the Department of Gynecology at the Women’s Hospital of Zhejiang University (Hangzhou, Zhejiang) for primary infertility. The patient was diagnosed with unilateral ovarian and fallopian tubal agenesis, without malformations of the uterus and urinary tract, during diagnostic laparoscopy and hysteroscopy. A literature review was conducted with the aim of determining the possible causes of these anomalies. However, the etiology of the adnexal anomaly remained unclear, although torsion or congenital defects were the most likely explanation. Therefore, the observations of the present study indicate that contralateral tubal pathologies may contribute to sterility.

## Introduction

Congenital agenesis of the unilateral adnexa is an uncommon condition that has rarely been described in the literature. In addition, the incidence of adnexal malformations is difficult to determine. It has been suggested to be 1:11,240 (1). Etiologies of ipsilateral ovarian and/or tubal agenesis remain unclear; however, a number of studies have conducted research into this area (1–4). A number of authors suggest that this abnormity is either a congenital malformation or a result of an torsion that occurred to the ovarian pedicle in birth, childhood or adult life (3,5). Adnexal agenesis has always been associated with malformations of the uterus and/or urinary tract. Unilateral absence of the adnexa without a uterine deformity is rarely reported. The majority of patients are asymptomatic and can be diagnosed incidentally following a laparoscopy or laparotomy for various gynecological or obstetric complications. The current study reports the case of a 26-year-old female who had been infertile for two years and had been diagnosed with unilateral agenesis of the ovary and fallopian tube during diagnostic laparoscopy and hysteroscopy. In the present study, the literature was reviewed in order to identify possible causes of these anomalies.

## Case report

A 26-year-old nulligravida was admitted to the Department of Gynecology at the Women’s Hospital of Zhejiang University (Hangzhou, Zhejiang) with a diagnosis of primary infertility. Informed consent was obtained from the patient for the present study. The patient had not conceived despite regular unprotected intercourse for two years. Menarche occurred at 12 years of age, and the menstrual cycle was regular with 27–28 day intervals and a 5–6-day menstrual period without dysmenorrhea. The overall health of the patient was good and there was no history of abdominopelvic surgery or pelvalgia. On physical examination, no surgical scars were observed. The external genitalia, vagina, cervix and uterus appeared normal on gynecological examination, and the results of sex hormone analysis were also normal. The patient’s husband submitted a semen analysis, which was within the normal limits. Transvaginal ultrasonography revealed that the uterus and ovaries (only the right ovary was visualized) were normal. Hysterosalpingography was performed in the Wenling Ruoheng Hospital (Wenling, China) additional local hospital, which revealed a normal uterine cavity; however, the fallopian tubes did not fill bilaterally; the hysterosalpingography was not repeated. Furthermore, genetic analysis revealed a normal karyotype (46,XX).

**Figure 1:**
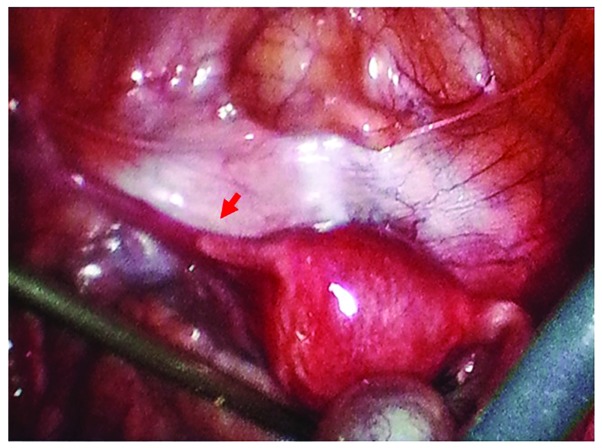
Laparoscopic view reveals a round ligament on the left side and a normal uterus, with no visible left tube or ovary, only a 2-cm tubal remnant. The right fallopian tube and right ovary (with a corpus luteum) were considered to be normal (arrow indicates tubal remnant).

Subsequently, a diagnostic laparoscopy and hysteroscopy were performed. During the hysteroscopy, the endometrial cavity was observed to be normal, as well as the left and right tubal ostia. The laparoscopy revealed a single, normal-sized uterus with a smooth surface. No adhesion between the uterus and the intestinal serosa, the cecum and the pelvic wall was observed. The rectouterine pouch was inspected and no ectopic tissues were identified. The left adnexa was not completely visualized; however, a 2-cm tubal remnant with an intact left round ligament was observed. The right fallopian tube, right ovary (with a corpus luteum) and right round ligament were found to be normal (Fig. 1). The broad ligaments were also normal without any adhesions. In addition, the peritoneal and omental surfaces were analyzed and no ectopic tissues or remnant structures were observed. Methylene blue chromopertubation did not result in spill from the right fallopian tube and the postoperative course was uneventful.

**Figure 2:**
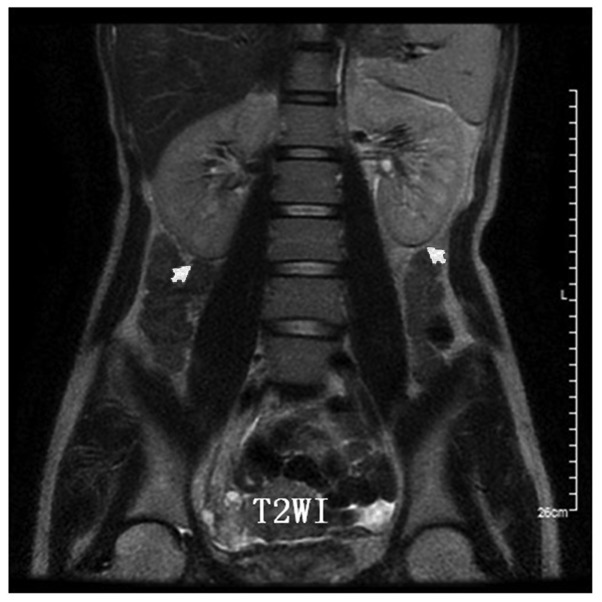
Abdominopelvic magnetic resonance imaging scan showing normal bilateral kidneys (white arrows indicating the normal kidneys).

Since adnexal agenesis often coexists with malformations of the urinary tract, abdominopelvic magnetic resonance imaging (MRI) was performed to investigate the urinary system. The MRI scan revealed that the kidneys and ureters were normal bilaterally, while the left ovary was unable to be imaged (Fig. 2).

Unilateral absence of the adnexa is rarely reported without a uterine deformity. Therefore, the present case report prompted a comprehensive literature review. Similar cases reported in the literature, describing unilateral agenesis or the absence of the ovary and fallopian tube without uterine anomalies, are presented in Table I. A total of 25 cases were identified, of which nine cases were diagnosed with primary infertility and seven cases had undergone normal deliveries. In particular, one patient was single, one had contraception by drugs, one had an extrauterine pregnancy and the fertility of the other six cases were not mentioned in the literature. Among the nine nulligravidas, three individuals had obstructed contralateral fallopian tubes, three patients had unobstructed tubes and the condition of the other three were not mentioned in the literature. In certain cases, dysmorphosis of the genital tract coexists with urinary tract anomalies. Two cases were found to be associated with ipsilateral absence of the kidney, while in six cases, urinary tract anomalies were not mentioned.

## Discussion

Congenital absence of the ovary is a rare condition, with one in every 11,240 individuals affected (6); however, the incidence may be higher as it is difficult to estimate the number of cases since the majority are asymptomatic and go unreported. All cases reported in the literature were diagnosed incidentally following a laparoscopy or laparotomy for various gynecological or obstetric complications. The first published case of unilateral ovarian absence was reported in 1923 (7). In recent years, several similar cases have been reported (1,4,8,9), which may be due to the widespread use of laparoscopy for diagnostic purposes; however, the total number of cases remains small.

Adnexal agenesis is often associated with malformations of the uterus and/or urinary tract, including a unicornuate uterus and unilateral renal agenesis (10). Congenital absence of the ovary may be accompanied with total or partial absence of the ipsilateral fallopian tube. However, unilateral absence of the adnexa without a uterine deformity is rarely reported. In the present case report, the uterus was considered normal in shape and structure during diagnostic laparoscopy and hysteroscopy. The literature was reviewed and a number of similar cases without uterine anomalies were identified (Table I). A number of studies have also evaluated the urinary tract in patients with ovarian agenesis (8,9). Among the cases listed, there were two patients with unilateral renal agenesis. In the present case, the results from the MRI scan revealed that the kidneys were normal.

The true etiology of ipsilateral ovarian and/or tubal absence has yet to be elucidated (1–3). The two most likely causes of ipsilateral ovarian and/or tubal absence may include an asymptomatic torsion of the adnexa with consequent organ ischemia and reabsorption (3,5,11), or a defect in the development of the Mullerian and gonadal structures (3,6,12) underlying vascular anomalies (11).

A number of studies have indicated that unilateral absence of the fallopian tube and ovary is the result of adnexal torsion with necrosis and resorption, which may occur antenatally or postnatally (3,5). Furthermore, it has been suggested that symptoms may be minimal or absent, although severe pain in the lower abdomen is a typical symptom of adnexal torsion (3). In 1974, Georgy and Viechnicki (13) reported a case with the absence of an ovary and uterine tube. This case supported the torsion hypothesis since a calcific ovary was situated in the Douglas cul-de-sac. The study by Uckuyu *et al* (14) also supported the torsion hypothesis since separated tubal and ovarian tissue remnants were observed in the abdominal cavity. In addition, Vaiarelli *et al* (4) reported a case where the ovary and ipsilateral fallopian tube were absent. The patient had presented with acute, transient right-sided pelvic pain 10 years previously, which was not diagnosed as adnexal torsion following medical attention. In retrospect, this acute pain may have represented torsion of the right adnexa.

A number of previous cases have demonstrated that unilateral ovarian absence coexists with teratomas on the great omentum or the uterine surface (15–17). Omental cystic teratomas are rare. The authors analyzed the torsion of the ovarian tumors that ruptured and parasitized on the greater omentum (15–17), and it was hypothesized that the incidence may be associated with embryonic developmental abnormalities. While this torsion hypothesis appears plausible, there is no evidence of its occurrence in the present case report. The patient had no history of unexplained abdominal pain, and during laparoscopic surgery, no ectopic tissues or remnant structures on the peritoneal or omental surfaces were observed. However, the absence of symptoms does not exclude the possibility of torsion antenatally.

Developmental abnormalities have been observed in the female reproductive tract, including the fallopian tubes, ovaries, uterus, cervix, vagina and external genitalia. Usually, abnormalities include organs that originate from the Mullerian ducts. In the sixth week of gestation, the bilateral Mullerian ducts migrate towards the midline, meet, form luminal structures, fuse and finally form the uterus and upper one-fifth of the vagina. Rostrally, the Mullerian ducts form fallopian tubes. Any disturbance in the migration, fusion or resorption of these ducts may result in a Mullerian anomaly (18). Paternoster *et al* (12) presented two cases of absent fallopian tubes, and hypothesized that partial or total unilateral defects of the paramesonephric duct were more common than aplasia of the two ducts. Therefore, a unicornuate uterus, one fallopian tube and one rudimentary or ectopic kidney indicates a defect in the development of all Mullerian structures.

In comparison to Mullerian duct-derived organs, congenital defects of the ovary are rare. Gonadal development depends on accurate germ cell migration, as well as appropriate formation of the urogenital ridge. These processes are regulated by multiple factors and genes (19), and a unilateral defect at any point during this process may prevent ovarian formation. Unilateral ovarian agenesis coexisting with an ipsilateral fallopian tube and a normal uterus is a complicated condition. It has been hypothesized that a defect localized to the region of the genital ridge and the caudal area of the Mullerian duct (5,20) reflects improper development of the urogenital ridge, which affects the development of the fallopian tube in that region. A number of studies have indicated that an inadequate blood supply during the descent into the pelvis of the caudal section of the paramesonephric duct may lead to adnexal agenesis (3,5,12); however, a clear developmental explanation for this malformation has not yet been elucidated. The patient in the current study presented for evaluation with a normal uterus and right adnexa observed during diagnostic laparoscopy and hysteroscopy; thus, excluding the possibility of a unicornuate uterus. Furthermore, the normal karyotype did not support the diagnosis of a chromosomal condition associated with the absence of the fallopian tube and ovary; for example, pure or mixed gonadal dysgenesis (46XY or 45X0/46XY).

A number of similar patients have been reported in the literature, with anatomic abnormalities observed during evaluations for primary infertility. It is unknown whether unilateral adnexal absence may be a cause of infertility. Several authors have hypothesized that unilateral adnexal absence does not diminish female fertility, particularly when the condition is not accompanied by a uterine malformation (9). Uckuyu *et al* (14) also investigated the function of the contralateral tube and concluded that unilateral agenesis is a possible factor in patients with infertility. Unilateral absence of the adnexa may reduce the probability of becoming pregnant; however, pregnancy remains possible if there is a functional fallopian tube. Previously, a patient with this condition was reported who had four normal pregnancies that resulted in normal vaginal deliveries (8). The patient presented in the current study was found to have an absent left adnexa and an obstructed right fallopian tube during laparoscopic examination, with no passage of methylene blue solution from the lumen of the right tube; however, the uterus and right ovary were normal. Previous studies have described contralateral occluded tubes (1,6,5). We hypothesized that contralateral tubal pathology may contribute to sterility. However, whether unilateral congenital tubal and ovarian anomalies affect the function of the other tube and the pelvic microenvironment remains unclear.

In conclusion, unilateral ovarian and fallopian tube agenesis is a rare condition. The true etiology of adnexal anomalies remain unclear, although torsion or congenital defects may be the most likely explanations. In addition, the observations of the present study indicate that contralateral tubal pathologies may contribute to infertility.

## Figures and Tables

**Table I:** Absence of ovaries and/or fallopian tubes with a normal uterus.

First author (reference)	Ovarian and/or tubal anomalies	Urinary anomaly	Fertility	Other notable observations
Elkington N (21)	Absent left tube and ovary	No	Normal delivery	
Pabuccu E (10)	Absent left tube and ovary	No	Primary infertility	
Vaiarelli A (4)	Complete absence of right ovary; 2-cm proximal stump of the right tube	NM	Primary infertility	History of acute pelvic pain 10 years previously
Gursoy AY (8)	Absence of left ovary and tube	Absence of left kidney	Normal delivery	
Eustace DL (3)				
Case 1	Absent right tube and ovary	No	Primary infertility	
Case 2	Absent right tube and ovary	NM	Normal delivery	
Sivanesaratnam V (6)				
Case 1	Absent left ovary and tube	No	NM	
Case 2	Absent right ovary and tube	No	Primary infertility	Blocked right tube
Mylonas I (5)				
Case 1	Absent right ovary and tube	No	Normal delivery	
Case 2	Absent right ovary and tube	No	Contraception by drugs	
Case 3	Absence right adnexa	No	Primary infertility	Blocked left tube
Muppala H (9)	Absent right ovary, tube and round ligament	Right renal agenesis	NM	Pyloric stenosis
Uckuyu A (14)				
Case 1	Absent left distal tubal segment, streak left ovary	No	Primary infertility	Unilateral tubal patency
Case 2	Absent right distal tubal segment, normal right ovary	No	Primary infertility	Unilateral tubal patency
Case 3	Twisted left tube, absent right ovary	No	Primary infertility	Unilateral tubal patency
Case 4	Left ovarian agenesis	No		
Tzitzimikas S (22)	Absence of the left ovary and the distal part of the ipsilateral tube	No	NM	
Gotti G (23)	Absent right ovary and tube	No	Extrauterine pregnancy	
Rapisarda G (1)	Absent left ovary and tube	NM	Primary infertility	Obstructed right tube
Sirisena LA (2)	Absent left ovary and distal tube	No	NM	
Georgy FM (13)	Absent left ovary and tube	No	NM	
Guan Q (15)	Absent right ovary and tube	NM	Normal delivery	Teratomas on the uterine surface
Liu Q (16)	Absent right ovary and tube	No	NM	Extraperitoneal huge serous cystadenoma
Ma CL (17)				
Case 1	Absent left ovary	NM	Normal delivery	Teratomas on the great omentum
Case 2	Absent left ovary and tube	NM	Normal delivery	Teratomas on the great omentum

NM, not mentioned.
